# Behavioral and neurological analyses of adult mice carrying null and distinct loss-of-receptor function mutations in protein tyrosine phosphatase receptor type Z (PTPRZ)

**DOI:** 10.1371/journal.pone.0217880

**Published:** 2019-06-13

**Authors:** Naomi Tanga, Kazuya Kuboyama, Ayako Kishimoto, Miho Kihara, Hiroshi Kiyonari, Toshio Watanabe, Akihiro Fujikawa, Masaharu Noda

**Affiliations:** 1 Division of Molecular Neurobiology, National Institute for Basic Biology (NIBB), Higashiyama, Myodaiji-cho, Okazaki, Aichi, Japan; 2 School of Life Science, The Graduate University for Advanced Studies (SOKENDAI), Higashiyama, Myodaiji-cho, Okazaki, Aichi, Japan; 3 Department of Biological Science, Graduate School of Humanities and Sciences, Nara Women’s University, Kita-uoya-nishi-machi, Nara, Japan; 4 Laboratories Laboratory for Animal Resource Development, RIKEN Center for Biosystems Dynamics Research, Minatojima Minami-machi, Chuou-ku, Kobe, Japan; 5 Laboratory for Genetic Engineering, RIKEN Center for Biosystems Dynamics Research, Minatojima Minami-machi, Chuou-ku, Kobe, Japan; 6 Cell Biology Center, Institute of Innovative Research, Tokyo Institute of Technology, 4529 Nagatsuta-cho, Midori-ku, Yokohama, Kanagawa, Japan; University Paris Diderot, FRANCE

## Abstract

Protein tyrosine phosphatase receptor type Z (PTPRZ) is preferentially expressed in the central nervous system as two transmembrane receptor isoforms PTPRZ-A/B and one secretory isoform PTPRZ-S. *Ptprz*-knockout mice lacking the expression of all three isoforms show behavioral, learning, and neurological abnormalities, including increased exploratory activities to novelty, deficits in spatial and contextual learning, and reduced responses to methamphetamine, relative to wild-type mice. To investigate whether PTPRZ isoforms play distinct physiological roles, we herein performed behavioral studies on two knock-in mouse lines: One expresses the catalytically inactive Cys-1930 to Ser (CS) mutants of PTPRZ-A/B, while the other generated in the present study expresses catalytically active mutants of PTPRZ-A/B lacking the negative regulatory PTP-D2 domain and C-terminal PDZ-binding motif (ΔD2) instead of wild-type PTPRZ-A/-B. In contrast to *Ptprz*-knockout mice, neither increased responses to novelty in the open field nor memory impairments in the inhibitory-avoidance task were observed in *Ptprz-*CS or *Ptprz*-ΔD2 mice. However, the effects of methamphetamine on locomotor activity were significantly weaker in *Ptprz*-KO mice and CS mutant mice than in wild-type mice, but were normal in ΔD2 mutant mice. Furthermore, microdialysis experiments revealed that methamphetamine-evoked dopamine release in the nucleus accumbens was reduced in *Ptprz*-KO mice and CS mutant mice. These results suggest that the extracellular region of PTPRZ, including the secretory isoform, is crucial for behavioral responses to novelty and the formation of aversive memories, whereas the PTPase activities of PTPRZ receptor isoforms are involved in regulating the dopaminergic system.

## Introduction

Protein tyrosine phosphorylation, one of the critical mechanisms for signal transduction, is reversibly regulated by protein tyrosine kinases (PTKs) and protein tyrosine phosphatases (PTPs). Receptor-like PTPs (RPTPs) are a diverse family of enzymes comprised of eight subfamilies [1]. Most RPTPs contain two tandem PTP domains intracellularly. Among them, catalytic activity is mainly retained in the first membrane-proximal domain (PTP-D1), and to a lesser extent or not at all in the second membrane-distal domain (PTP-D2). In the case of R5 subfamily member including PTPRZ, the PTP-D2 domain is a catalytically inactive PTPase domain and is considered to function in the formation of the inactive “head-to-toe” dimers [2].

Protein tyrosine phosphatase receptor type Z (PTPRZ, also called PTPζ) is a member of the R5 RPTP subfamily together with PTPRG (PTPγ). PTPRZ and PTPRG structurally resemble each other, whereas their tissue distribution markedly differs: PTPRZ is predominantly expressed in the central nervous system (CNS), whereas PTPRG is expressed ubiquitously. The *Ptprz* gene encodes three major splicing isoforms: the long receptor isoform, PTPRZ-A consists of a carbonic anhydrase (CAH)-like domain, fibronectin type III-like domain followed by a spacer region, a membrane-spanning region, and cytoplasmic tandem PTP domains with a canonical PDZ-binding motif (-Ser-Leu-Val) at the carboxyl terminal end; the short receptor isoform, PTPRZ-B has a deletion in the extracellular spacer region from PTPRZ-A; and the secretory isoform, PTPRZ-S corresponds to the extracellular portion of PTPRZ-A. The two receptor isoforms have been further classified into two submembers, “conventional PTPRZ-A or -B” and “exon 16-deleted PTPRZ-A or -B(Δex16)”, respectively [3]; in the present study, we refer to both as “PTPRZ-A or-B” collectively.

Three lines of knockout mice deficient in Ptprz have been generated with different strategies by three independent groups [4–6], all of which are grossly normal. *Ptprz*-null knockout (*Ptprz*-KO) mice, which were generated in our laboratory, lacked the expression of all three isoforms [4]. Our *Ptprz*-KO are apparently healthy and exhibit no obvious morphological abnormalities in the brain at the adult stage [4]; however, several phenotypic (functional) alterations have been identified [4, 7–12]: They show the early onset of oligodendrocyte differentiation and myelination in the developing brain [7]. They show reduced disease severity for experimental autoimmune encephalomyelitis [7] and accelerated remyelination in the cuprizone model [8, 9]. In contrast, another knockout line reported by Harroch et al. exhibited a fragility of myelin in the CNS [5] and impaired remyelination [13], suggesting a positive role of Ptprz in oligodendrocyte survival and in recovery from demyelinating disease. Behavioral and neurological studies of our *Ptprz*-KO mice revealed impairments in spatial and contextual learning as well as memory functions [11, 14]. As for behavioral phenotypes, the knockout mice reported by Lafont et al. exhibited impaired working memory functions, an altered motor coordination, and reduced responses to moderate thermal and tactile stimuli [15]. Of note, we found that gastric mucosal cells in peripheral tissues express the PTPRZ-B isoform, but at lower levels [12]. *Ptprz*-KO mice are resistant to gastric ulceration caused by VacA, a cytotoxin secreted by *Helicobacter pylori* [12], suggesting that PTPRZ is a functional receptor for VacA.

All three isoforms expressed in the brain have chondroitin sulfate chains on their extracellular portion [16–18]. The chondroitin sulfate moiety is essential for achieving high-affinity ligand binding [19–21]. Regarding the receptor isoforms of PTPRZ-A and -B, the binding of endogenous ligand molecules, such as pleiotrophin (PTN)/heparin-binding growth-associated molecule (HB-GAM) [9, 19, 22, 23], midkine (MK) [20], and interleukin-34 (IL-34) [21], to the extracellular portion inactivates cytoplasmic PTPase by inducing receptor clustering [23]. Signaling from the ligand to PTPRZ receptors is regarded as the forward signal. Secretory PTPRZ-S, also known as phosphacan/6B4 proteoglycan/DSD-1, is a major chondroitin sulfate-proteoglycan in the CNS [24–26]. PTPRZ-S is one of the extracellular matrix (ECM) and perineuronal net (PNN) components, serving as a substratum for multiple cell adhesion molecules, including F3/contactin [27, 28]. The binding signal from the extracellular region of PTPRZ isoforms to (unknown) receptors on different cells has been regarded as a reverse signal.

These findings suggest that the receptor and secreted isoforms play distinct, but complementary roles in regulating development and functions; however, the specific contributions of individual PTPRZ isoforms have remained unclear. In the present study, we generated and characterized the neurological phenotypes of knock-in mutant mice carrying targeted loss-of-specific functions or domains of PTPRZ receptors relative to *Ptprz*-KO mice.

## Results

### Generation of *Ptprz-*ΔD2 knock-in mutant mice

**Fig 1:**
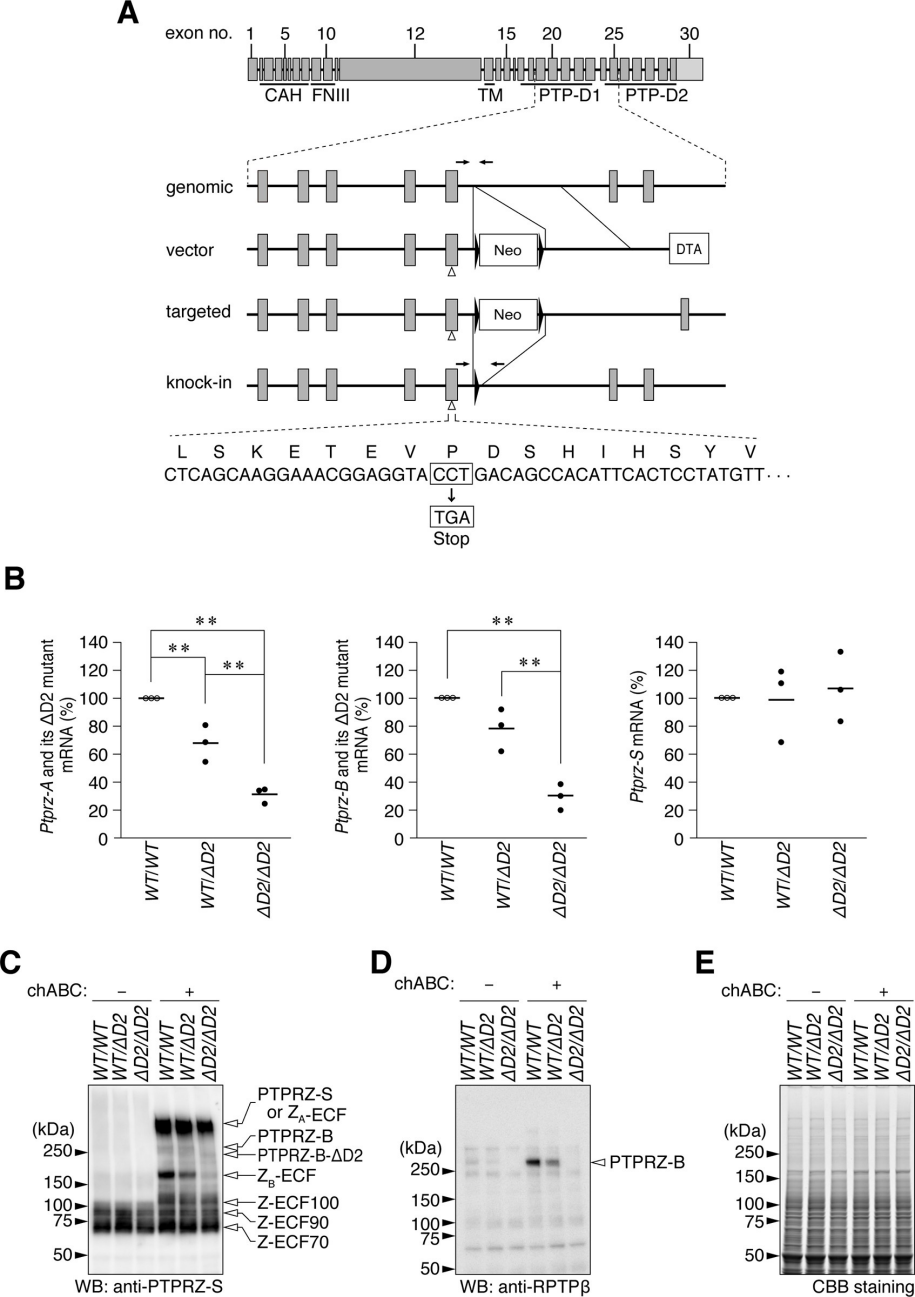
*Ptprz*-ΔD2 knock-in mouse. (**A**) Strategy to generate a Pro-1998 to termination (ΔD2) knock-in in the *Ptprz* gene. Schematic representation of the exon/intron structure of the *Ptprz* gene. Each box indicates an exon with the exon number, and the final exon 30 containing the 3'-non-coding sequence (light gray). Horizontal arrows indicate PCR primer sites for mouse genotyping. CAH, carbonic anhydrase-like domain; FNIII, fibronectin type III domain; TM, transmembrane region; PTP-D1 and -D2, tyrosine phosphatase domain 1 and 2. Neo, neomycin-resistance gene cassette; DTA, diphteria toxin A gene cassette. Filled triangles show *loxP* sites. (**B**) Quantitative RT-PCR. The mRNA expression levels of *Ptprz-A* and *Ptprz-B* (total of both wild-type and ΔD2 mutant forms) and *Ptprz-S* in adult brain tissues were measured using the respective primer sets shown in S1 Fig. They were normalized to *Gapdh* expression, and plotted as relative values to *wt*/*wt* mice (*n* = 3 individual mice per group). *wt*/*wt*, homozygous for the wild-type allele; *wt*/Δ*D2*, heterozygous; and Δ*D2*/Δ*D2*, homozygous for the ΔD2 mutant allele. There were significant effects of genotype on the expression levels of *Ptprz-A* (*F*_(2, 6)_ = 55.663, *P* = 0.000) and *Ptprz-B* (*F*_(2, 6)_ = 37.680, *P* = 0.000), but not *Ptprz-S* (*F*_(2, 6)_ = 0.006, *P* = 0.874) by a univariate analysis of variance (UNIANOVA). **, *P* < 0.01, significant difference between the indicated groups by Tukey’s *post-hoc* HSD test. (**C, D**) Western blot analyses of brain extracts treated with (+) or without (-) chondroitinase ABC (chABC). In brain tissues, PTPRZ-A, -B, and -S and their processed derivatives (Z_A_-ECF and Z_B_-ECF) were highly modified with chondroitin sulfate chains, and, therefore, the chABC treatment beforehand was necessary for resolving their core proteins by SDS-PAGE [17, 30]. Anti-PTPRZ-S (*C*) and anti-RPTPß (*D*) recognize the extracellular region of all three PTPRZ isoforms, and the epitope on the C-terminal part of the PTP-D2 domain, respectively [17]. (**E**) CBB staining of samples to verify their protein amounts applied to each lane.

We recently generated a targeted knock-in mouse line carrying a Cys to Ser mutation in PTP-D1 (PTPase-inactive CS mutant) [29]. According to the inactive “head-to-toe” dimerization model [2], D2-truncated mutants of PTPRZ-A/B are considered to be constitutively active PTPase. In the present study, we newly generated a *Ptprz*-ΔD2 knock-in mouse line in which Pro (1998) at the carboxy-terminal end of PTP-D1 was substituted to a termination codon (Fig 1A). *Ptprz*-CS mice and *Ptprz*-ΔD2 mice were backcrossed over ten generations with the C57BL/6J (WT) strain as well as *Ptprz*-KO mice. Homozygous ΔD2 mice were healthy and exhibited apparently normal growth and reproduction. DNA sequencing of cDNA clones prepared from homozygous *Ptprz*-ΔD2 mouse brains validated the successful knock-in of the desired point mutation (S1A Fig). Quantitative reverse transcription-PCR (qRT-PCR) analyses revealed that the expression levels of mRNAs for the ΔD2 mutants of PTPRZ-A and -B were significantly lower than those of their normal forms in wild-type mice (Fig 1B, left and center; wild-type > heterozygous > homozygous), whereas the level of *Ptprz-S* mRNA was not significantly different (Fig 1B, right).

Western blotting showed that the expression amount of the ΔD2 mutant form of PTPRZ-B was lower than that of wild-type PTPRZ-B in brain extracts (Fig 1C), consistent with the results of qRT-PCR. In the brain, large amounts of PTPRZ proteins are proteolytically processed by metalloproteinase [17] or plasmin [30]. The core protein of PTPRZ-A at 380 kDa was only negligibly detected because of proteolytic cleavage into the whole extracellular fragment, Z_A_-ECF [17, 30]. PTPRZ-A/B receptor isoforms undergo metalloproteinase- or plasmin-mediated cleavage, releasing various extracellular fragments in the normal brain [17, 30]. Since Z_A_-ECF is indistinguishable from PTPRZ-S based on their mobilities in SDS gel electrophoresis, it was not possible to quantify its amount at the protein level. Consistent with the reduction of the receptor isoforms with the ΔD2 mutation, the amount of its whole extracellular fragment, Z_B_-ECF at 180 kDa, was also reduced by the ΔD2 mutation. No apparent changes were observed in Z-ECF-100, -90, or -70, which corresponded to the fragments of the extracellular portion of PTPRZ-A/-B produced by plasmin cleavage. To discriminate two close bands of PTPRZ-B and PTPRZ-B-ΔD2, we also analyzed the blot with another antibody (anti-RPTPß), which recognizes an epitope on the C-terminal part of the PTP-D2 domain of PTPRZ-A/-B receptor isoforms [17], but does not detect the ΔD2 mutant. The Western blotting with anti-RPTPß indicated no protein expression of the native full length form of PTPRZ-B products in the homozygous, and verified that the band of a slightly lower size than PTPRZ-B corresponds to the ΔD2 mutant form (Fig 1D). Here, we should note that the ΔD2 mutant is an constitutively active PTPase in living cells [31].

### Open-field behavior and inhibitory avoidance learning of *Ptprz-*CS and *Ptprz*-ΔD2 knock-in mice

**Fig 2:**
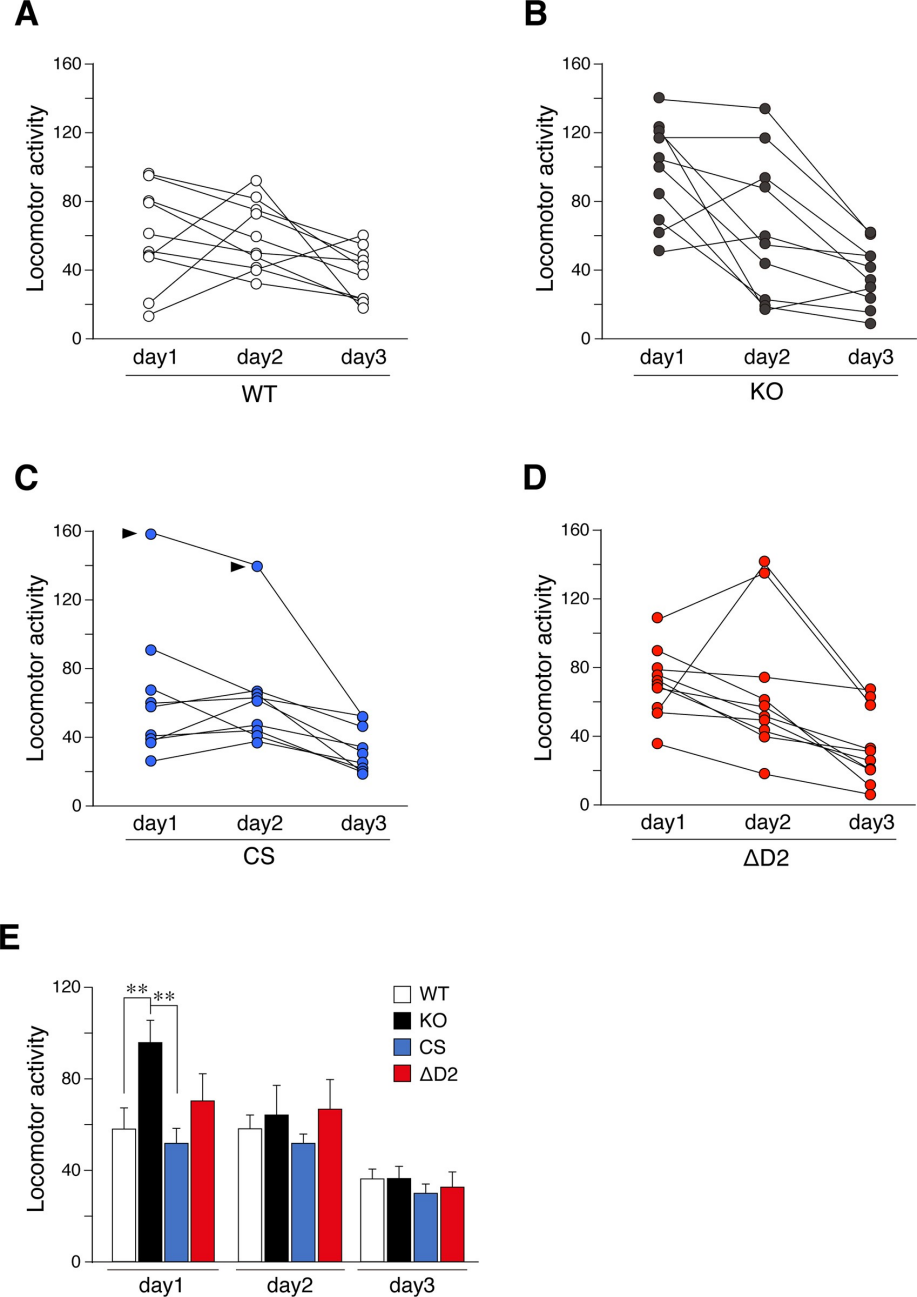
Open-field test. **(A to E)** Horizontal activities in the open field. Mice were exposed to an open field environment for 5 min per day for 3 consecutive days. Scatter plots show individual values on each day for wild-type (*A*), *Ptprz*-KO (*B*), CS knock-in (*C*), and ΔD2 knock-in (*D*) mice (*n* = 9–10 animals per group). Two values that were obtained from one CS-knock-mouse were regarded as outliners by Grubb's test (pointed by arrowheads). Therefore, the results from the CS knock-in mouse were eliminated for the reliable verification, and the trimmed data set was shown as a bar graph with the mean and stander error (SE) (*E*). There were significant effects of day (*F*_(2, 68)_ = 30.665, *P* = 0.000), and interactions of between day and genotype (*F*_(6, 68)_ = 2.363, *P* = 0.049), but no significant effects of genotype (*F*_(3, 34)_ = 1.889, *P* = 0.170) by two-way mixed design ANOVA. **, *P* < 0.01, significantly different between the indicated groups by *posthoc* Tukey HSD test.

**Fig 3:**
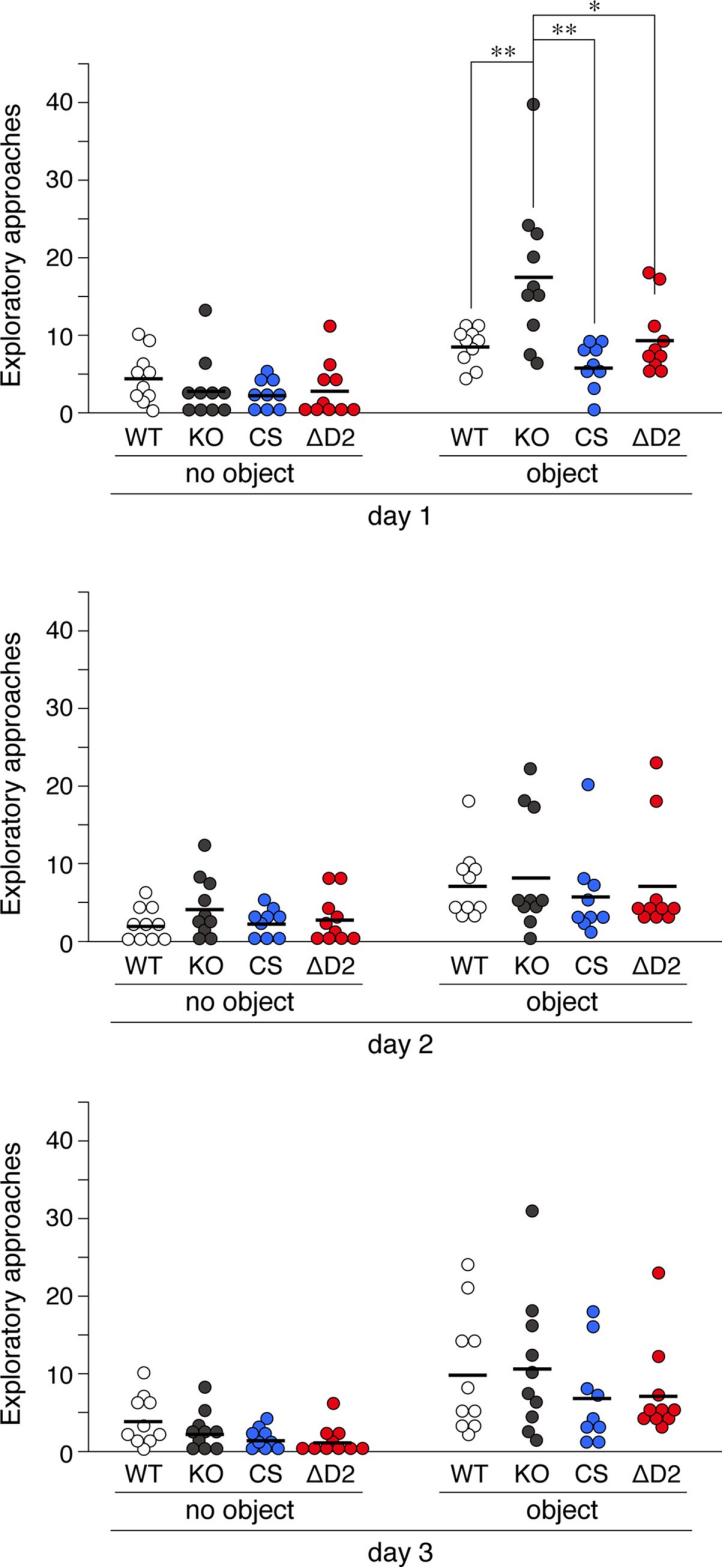
Novel object exploration test. Tests were performed in the same open field, first in the absence of an object for 9 min and then in its presence at the center for 9 min for 3 consecutive days. The scatter plot shows the individual values of exploratory activities with their mean (*n* = 9–10 animals per group). There were significant effects of object on all three days (Day 1, *F*_(1, 35)_ = 71.992, *P* = 0.000; Day 2, *F*_(1, 35)_ = 22.917, *P* = 0.000; Day 3, *F*_(1, 35)_ = 38.217, *P* = 0.000), and significant effects of genotype on day 1, but not day 2 or 3 (Day 1, *F*_(3, 35)_ = 4.175, *P* = 0.013; Day 2, *F*_(3, 35)_ = 0.440, *P* = 0.726; Day 3, *F*_(3, 35)_ = 1.049, *P* = 0.383) by a two-way mixed design ANOVA. *, *P* < 0.05; **, *P* < 0.01, significant difference between the indicated groups by Tukey’s HSD test.

Adult (4 to 6 months old) male mice were used in subsequent experiments. In the open field test, *Ptprz*-KO mice showed significantly stronger horizontal activity than wild-type mice on day 1, but not thereafter (Fig 2), indicating an enhanced response to a novel environment, as reported previously [32]. However, *Ptprz-*CS and *Ptprz*-ΔD2 mice showed similar responses to wild-type mice in the open-field test under the same conditions. In the novel object exploratory test, exploratory activity to a novel object was greater by *Ptprz*-KO mice than by wild-type mice on day 1 (Fig 3), whereas *Ptprz*-CS and *Ptprz*-ΔD2 mice showed no alterations, indicating that this activity is independent of PTPase activity.

**Fig 4:**
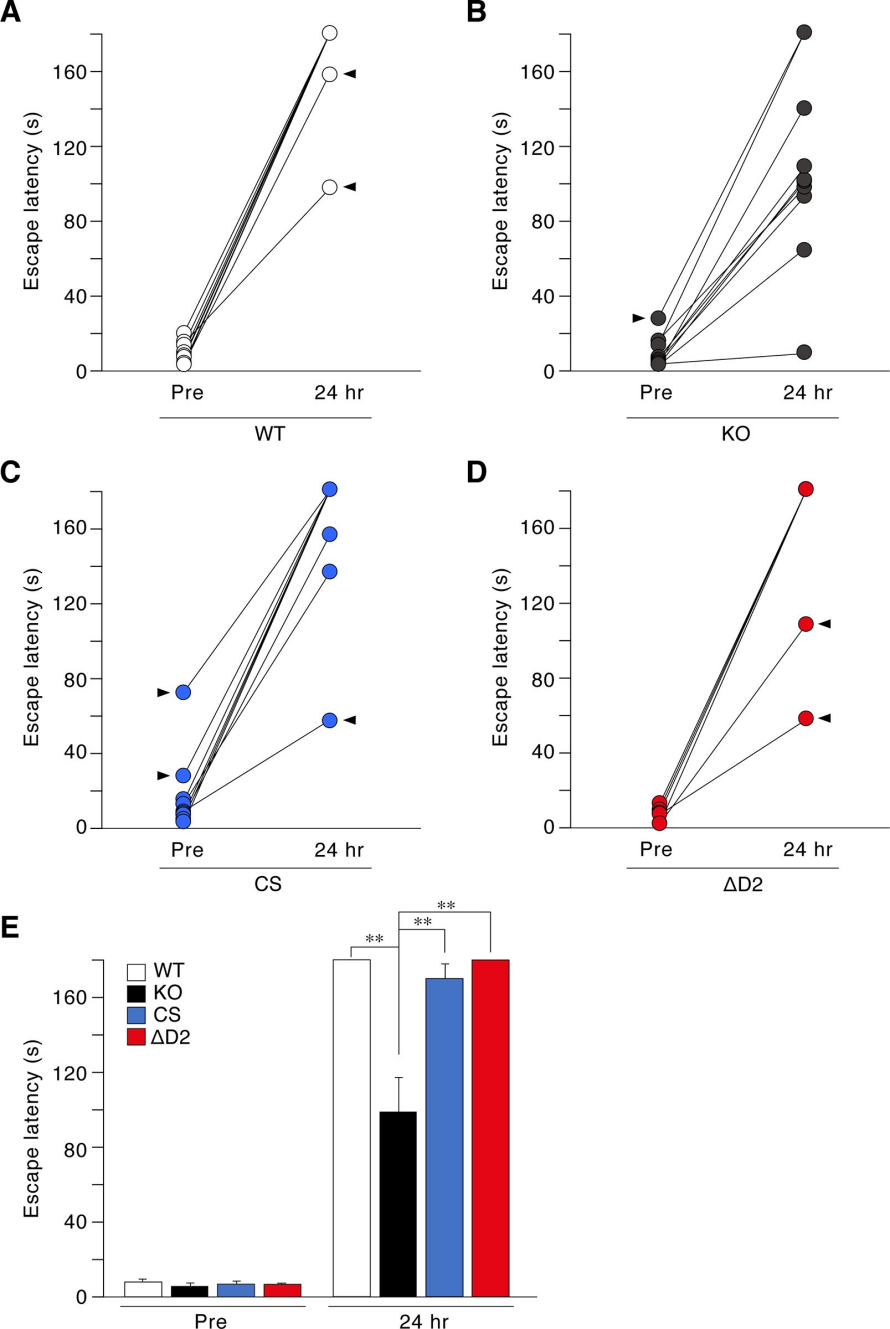
Aversive learning. **(A to E)** A step-through inhibitory avoidance test. Mice were placed in the light compartment, and their escape latency to enter the dark compartment was measured before receiving footshocks (Pre) and 24 hrs after footshock conditioning. Scatter plots show individual values of wild-type (*A*), *Ptprz*-KO (*B*), CS knock-in (*C*), and ΔD2 knock-in (*D*) mice (*n* = 10 animals per group), in which eight values obtained from eight mice were outliners by Grubb's test (pointed by arrowheads). After removing the data of the eight mice, the trimmed data set was shown as a bar graph with the mean and SE (*E*). There were significant effects of training (*F*_(1,28)_ = 1053.244, *P* = 0.000), genotype (*F*_(3, 28)_ = 17.920, *P* = 0.000), and interactions of between training and genotype (*F*_(3, 28)_ = 18.842, *P* = 0.000) by two-way mixed design ANOVA. **, *P* < 0.01, significantly different between the indicated groups by *posthoc* Tukey HSD test.

We then performed a step-through inhibitory avoidance task that depends on the functions of the hippocampus and amygdala [33, 34]. Mice placed in the bright chamber exhibit behavior to immediately escape to the dark compartment. Escape latency did not significantly differ among the genotypes (“*Pre*” in Fig 4). Mice then received footshocks in the dark compartment, and were left there for 2 min to learn the footshock-context association. This learning session was repeated until each mouse showed a predetermined latency of 3 min. The number of repetitions required to reach the predetermined time did not significantly differ among the genotypes (Mean ± standard deviation; WT = 3.1 ± 0.99, KO = 2.8 ± 0.79; CS = 2.8 ± 1.03, ΔD2 = 3.0 ± 0.67; UNIANOVA: *F*_*(*3, 36)_ = 0.288, *P* = 0.834), indicating the normal acquisition of aversive conditioning in all genotypes (groups). Memory retention was then tested 24 h later. Only *Ptpr*z-KO mice showed a shorter latency than wild-type, CS, and ΔD2 knock-in mice (“*24 hr*” in Fig 4). These results indicated that the catalytic activity of PTPRZ was not essential for a normal novelty response or aversive learning.

### Reduced responses to METH in CS mutant mice

**Fig 5:**
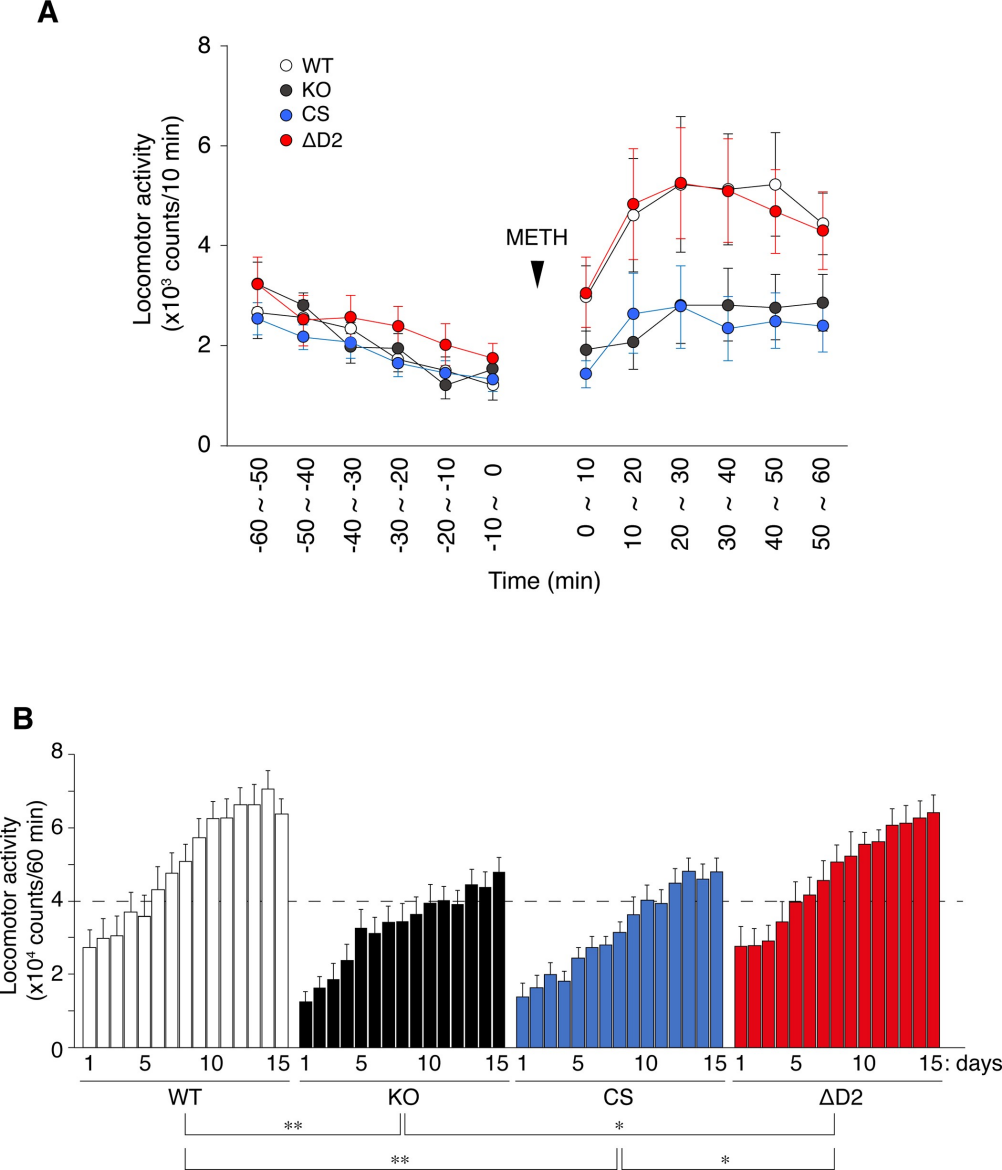
Locomotor response to METH administration. (**A**) Locomotor response to a single METH injection. After habituation to the ambulation chamber, mice were administered METH (1 mg/kg) and this was taken as time zero. Locomotor activity, as indexed by the number of sensor counts, was automatically recorded at 10-min intervals. The graph shows the mean values with standard errors (SE) on day 1 (*n* = 13 per group). (**B**) METH-induced locomotor sensitization. Mice were treated daily with METH (1 mg/kg), and locomotor activity was recorded for 60 min. The graph shows cumulated values for 60 min after the METH injection with the mean of sensor counts with SE on days 1 to 15 (*n* = 13 per group). There were significant effects of genotype (*F*_(3, 48)_ = 7.151, *P* = 0.000) by a two-way mixed design ANOVA. *, *P* < 0.05; **, *P* < 0.01, significant difference between the indicated groups by the Bonferroni *post-hoc* test.

Methamphetamine (METH) is a drug of abuse that induces an increase in locomotor activity in rodents, and repeated METH exposure causes adaptive changes in central dopaminergic systems, which may underlie the mechanism of locomotor sensitization to METH [35–37]. We previously reported that *Ptprz-*KO mice showed significantly weaker locomotor responses to a single and repeated injections of METH at a dose of 1 mg/kg than wild-type mice (whereas the locomotor response to 3 mg/kg of METH did not significantly differ) [32]. Under the same experimental conditions, *Ptprz*-CS mice and *Ptprz*-KO mice similarly showed weaker responses to METH than wild-type mice (Fig 5A). Furthermore, METH-induced locomotor sensitization was significantly reduced in *Ptprz*-CS and *Ptprz*-KO mice. However, *Ptprz-*ΔD2 mice showed no alterations (Fig 5B). These results indicated that these reductions in response are due to PTPase activity.

**Fig 6:**
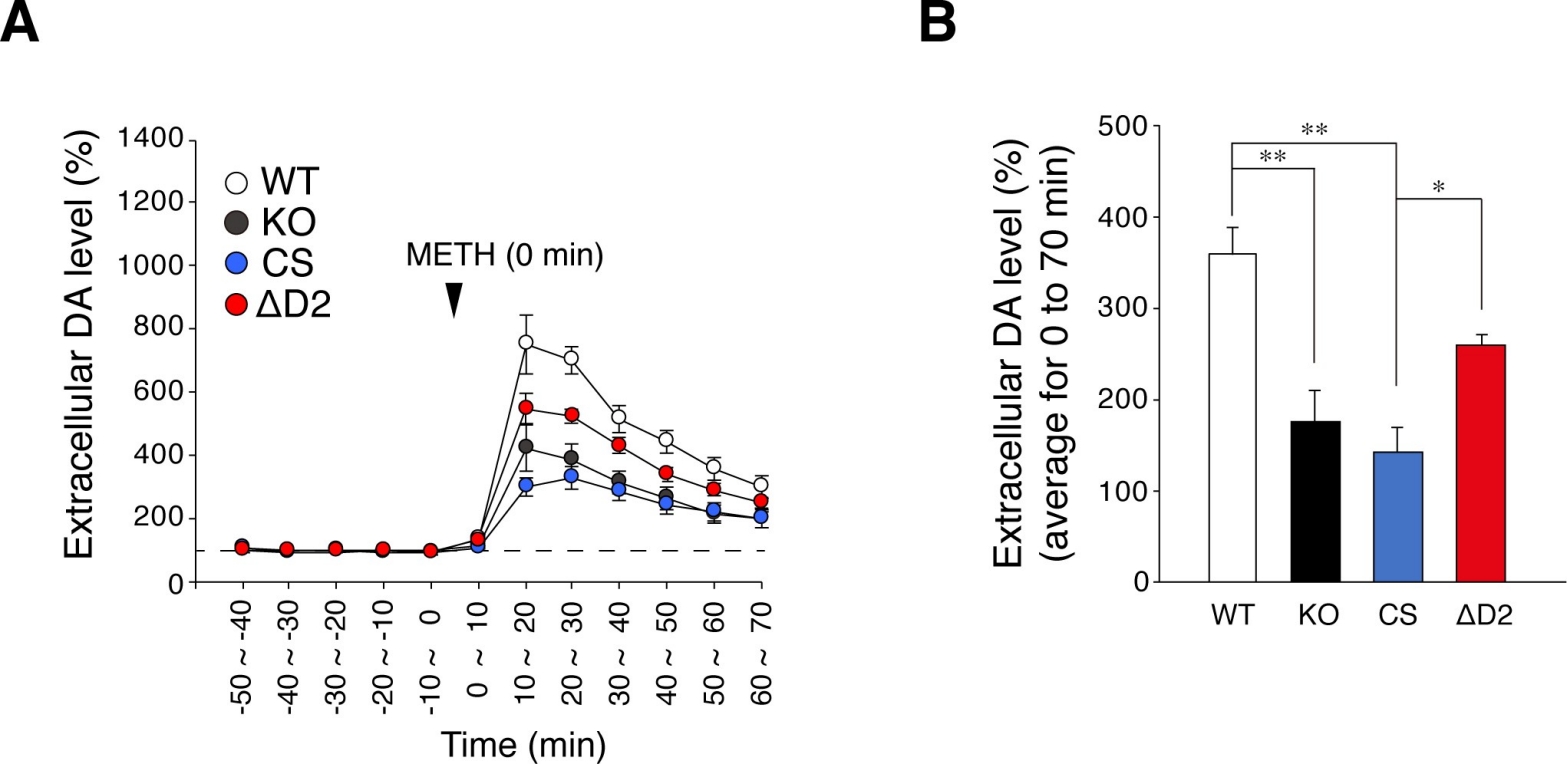
METH-evoked dopamine release in the nucleus accumbens. (**A, B**) *In vivo* microdialysis measurements of extracellular dopamine in the nucleus accumbens in freely moving mice. Perfusates were automatically collected at 10-min intervals and DA levels were measured. The plot shows the mean ± SE of DA levels in perfusates (*n* = 7 per group) (*A*). Dopamine levels were expressed as the percentage of averaged basal values (three points, -30 ~ -20 min, -20 ~ -10 min, and -10 ~ 0 min). (**B**) Summary of METH-evoked dopamine release. The graph shows the mean with SE of extracellular DA levels from 0 ~ 10 min to 60 ~70 min after the METH treatment. There were significant effects of genotype (*F*_(3, 24)_ = 13.319, *P* = 0.000) by UNIANOVA. *, *P* < 0.05; **, *P* < 0.01, significant difference between the indicated groups by Tukey’s *post-hoc* HSD test.

METH-induced hyper-locomotion is caused by increases in extracellular dopamine levels in the terminal field of the mesolimbic dopamine pathways, particularly in the nucleus accumbens [35]. *Ptprz*-KO mice showed a reduction in METH-induced dopamine efflux in the nucleus accumbens [32]. Microdialysis measurements revealed that METH-evoked dopamine release in the nucleus accumbens was also lower in *Ptprz-*CS mice than in wild-type mice (Fig 6). METH-induced dopamine release was slightly lower in *Ptprz-*ΔD2 mice than in wild-type mice (*p* = 0.063 vs wild-type mice by Tukey’s *post-hoc* test), but was significantly higher than that in *Ptprz*-CS mice. These results indicated that the loss of PTPase activity is associated with reduced responses to METH.

## Discussion

**Fig 7:**
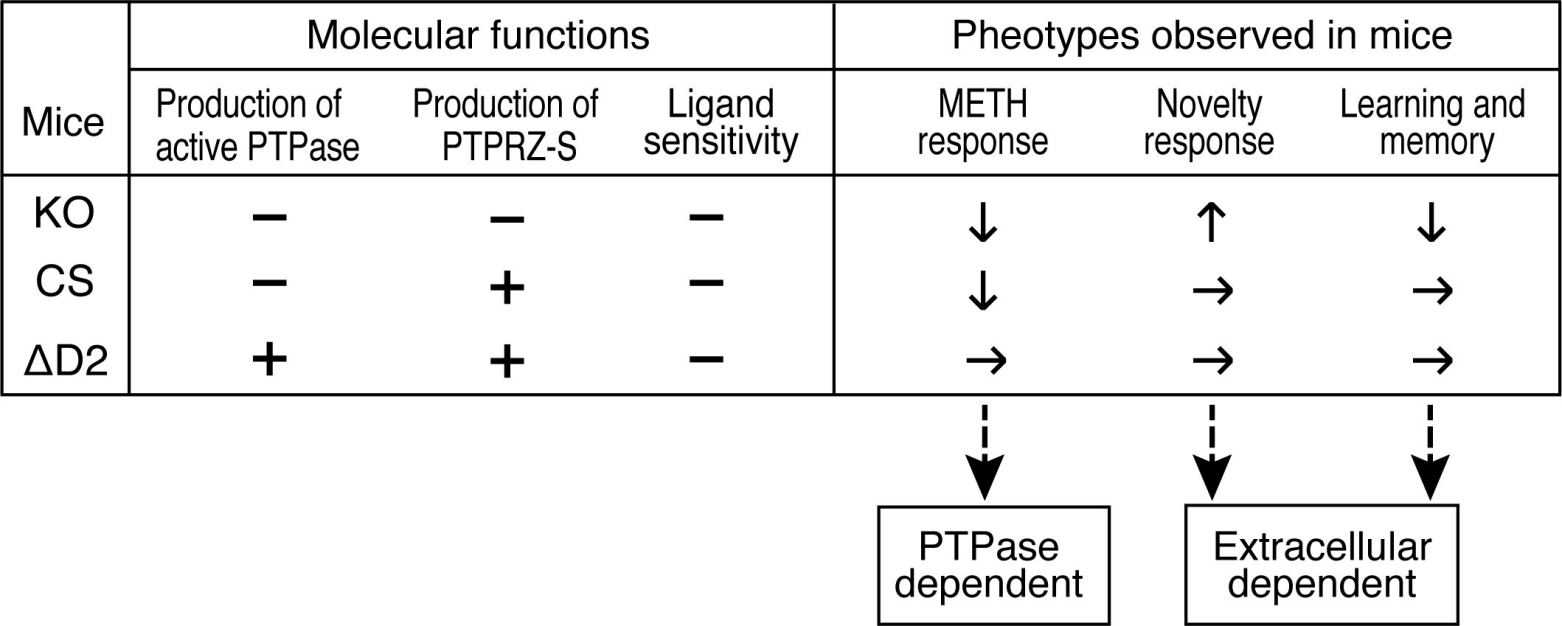
Summary of *Ptprz*-KO, and *Ptprz*-CS and ΔD2 knock-in mutant mouse phenotypes. Molecular function is maintained (+) or disrupted (–). The phenotype is evident (Up/increase or down/decrease with arrows) or not (horizontal arrows). Ligand (PTN) sensitivity means the ligand-induced PTPase inactivation of PTPRZ receptors. Distinct physiological roles of PTPRZ isoforms might be a reason to explain the discrepancy in the myelination phenotype between our *Ptprz*-KO mice [7, 8, 29] and another KO mouse line [13], which is mentioned in the introduction section. Because the latter KO line was generated by the replacement of an exon encoding a portion of the extracellular CAH domain with a pgk-neo cassette [5], which may result in unexpected expression of an aberrant extracellular fragment of PTPRZ.

The present results revealed that *Ptprz*-CS mice and *Ptprz*-KO mice showed attenuated METH-induced locomotor activations with reductions in dopamine efflux in the nucleus accumbens, whereas increased responses to novelty and impaired memory retention in the aversive learning task were only observed in *Ptprz*-KO mice. To the best of our knowledge, the present study is the first to suggest that PTPRZ isoforms may be involved in distinct physiological functions in the brain; therefore, we considered that the enhanced response to low METH responsiveness should be due to the loss of the forward signal by the PTPase activity of PTPRZ receptors, whereas novelty and impaired memory retention appeared to be due to the loss of the reverse signal by the extracellular region, including the secretory isoform, PTPRZ-S (see Fig 7).

*Ptprz*-KO mice exhibit maturation-dependently enhanced LTP in the CA1 region in hippocampal slices and impaired hippocampal-dependent learning [11, 14]. This phenotype is slightly inconsistent with the finding that mice lacking PTN, an inhibitory ligand of PTPRZ receptors, exhibit enhanced hippocampal LTP with learning impairments [38]. However, CS and **Δ**D2 mutant mice both displayed normal memory in the aversive learning task, indicating that the loss of PTPRZ activity or interactions with synaptic PDZ domain-containing proteins do not confer the hippocampal phenotypes found in *Ptprz*-KO mice. PTN may modulate hippocampal synaptic plasticity through receptors other than PTPRZ, including syndecan3 (SDC3), a transmembrane heparan sulfate proteoglycan predominantly expressed in the hippocampus of adults. *Sdc*3-knockout mice exhibit impaired performance in tasks assessing hippocampal functioning together with enhanced LTP in the hippocampal CA1 region [39]. Enhanced LTP in *Sdc3*-knockout mice is not responsive to PTN, which inhibits LTP in wild-type animals [39].

Normal aversive learning in CS and ΔD2 knock-in mutant mice may emphasize the crucial role of secretory PTPRZ-S in the regulation of hippocampal function. PTPRZ-S acts as a ligand for F3/contactin1 (CNTN1) [27, 28]. Whole-cell recordings from the CA1 pyramidal cells of *Cntn1*-knockout mice at P15 to P17 revealed their impaired paired-pulse facilitation (PPF) and long-term depression (LTD) with normal LTP and synaptic morphology [40]. Notably, in *Cntn1* knockout mice, the abnormal distribution of anti-phosphacan (3F8) immunoreactivity was detected in synapse-rich areas, the stratum radiatum and stratum lacunosum moleculare, in the hippocampus [40]; however, the effects of the knockout of *Cntn1* on hippocampal learning ability in adult mice has not been examined because of early postnatal lethality (by P18) [41]. Transgenic mice overexpressing CNTN1 are functionally normal at 5 months of age, but show enhanced LTP in the CA1 and improved spatial and object recognition memory at 12 months of age [42], which appears to be consistent with the maturation-dependent (>13 weeks old) hippocampal alteration in *Ptprz*-KO mice [14]. PTPRZ-S may function as a ligand of the CNTN1 receptor (or receptor complex) in the hippocampus. Future studies on mice selectively lacking the *Ptprz-S* isoform are needed to clarify this point and elucidate the signaling pathways involved in the regulation of hippocampal functions as the reverse signal.

PTPRZ receptor isoforms are expressed in the majority of midbrain dopamine neurons, and *Ptprz*-KO mice showed reduced responses to METH [32]. The present study revealed that CS knock-in mice also showed weaker locomotor responses and lower mesolimbic dopamine release to METH. Furthermore, no significant changes were observed in the expression level of tyrosine hydroxylase (TH), the rate-limiting enzyme for DA biosynthesis, in striatal extracts in the mouse groups (S2 Fig). These results strongly suggest that the PTPase activities of PTPRZ receptors positively affects the stimulant effects of METH as the forward signal. Mice deficient in RET tyrosine kinase show reduced dopamine transporter (DAT) activity and diminished behavioral responses when exposed to cocaine [43], suggesting that the loss of PTPs that counteract RET kinase activity reduces METH responsiveness. It is important to note that mice carrying a heterozygous knockout mutation in the *Ptprd* gene encoding PTPRD take longer to establish the self-administration of cocaine than wild-type littermate controls [44]. The same study also revealed that 7-butoxy illudalic acid analog (7-BIA), which inhibited the phosphatase activity of PTPRD *in vitro*, reduced cocaine rewards in self-administration and conditioned place-preference tests [44]. Although the downstream substrate proteins for PTPRD involved in cocaine rewards or their relevance to the inhibition potency of 7-BIA for PTPRD activity *in vivo* remain unclear, these findings suggest the loss of PTPs counteracting GDNF-RET signaling is also connected to reductions in METH responsiveness. It will be of interest to elucidate the relationship and crosstalk between RPTPs, including PTPRZ, and RPTKs, such as RET.

The inhibitory PTPRZ ligand, PTN is up-regulated after a single administration of amphetamine in the nucleus accumbens [45], and it (a relatively high dose of 10 mg/kg) induces conditioned place preference in *Ptn*-KO and wild-type mice at similar levels, whereas, 5 days after the last amphetamine injection, *Ptn*-KO mice, but not wild-type mice, still show a preference for the amphetamine-paired side [46]. However, this study identified enhanced amphetamine-induced astrocytosis in the striatum of *Ptn*-KO mice [46], suggesting that PTN limits amphetamine-induced reward effects by ameliorating neurotoxic effects. Besides endogenous PTPRZ ligands, it was recently reported that mice treated with MY10, a small-molecule inhibitor of PTPRZ, drank less ethanol than controls, and the MY10 treatment blocked ethanol-conditioned place preference; however, the effect, efficacy, and specificity of MY10 on PTPRZ phosphatase activity have not yet been demonstrated [47]. Ligand (PTN)-induced PTPase inactivation through “head-to-toe” dimerization [48] is expected to be abolished in ΔD2 mutant mice. Future studies using these mutant mice will be valuable for clarifying the involvement of PTN-PTPRZ signaling in METH or amphetamine responses as well as in the development of abused drug dependence.

## Materials and methods

### Ethics statement and experimental animals

All experimental animal protocols used in the present study were approved by the Institutional Animal Care and Use Committee of the National Institutes of Natural Sciences (approval numbers: 12A078, 13A172, 14A150, 15A095, 16A147, and 17A023), and the RIKEN Kobe Branch (approval number: A2001-03-72), Japan.

*Ptprz*-KO mice [4] and *Ptprz*-CS mice (ref) were backcrossed with the inbred C57BL/6J strain (CLEA Japan) for more than ten generations. Mice were housed under specific pathogen-free (SPF) conditions at a constant room temperature (23°C) and 50–55% humidity with a 8:00 to 20:00 light cycle. Four to 5 weeks after birth, 3 to 4 sex-matched mice were housed in a plastic cage (cage size: 12 × 21 × 12.5 cm) with paper-chip bedding, and food and water were provided *ad libitum*.

Adult male mice (4 to 6 months old) were used in the present study. Mice were handled gently to minimize stress. Surgeries for implanting a guide cannula for brain microdialysis were performed under isoflurane anesthesia, 2% lidocaine cream was applied to the incision site after surgery for acute pain relief, and all efforts were made to minimize suffering. Behavioral experiments were performed during the light period (between 9:00 and 17:00) by at least two different individuals under blind conditions. The method of sacrifice used for the experimental mice is decapitation with surgical scissors.

### Generation of *Ptprz-*ΔD2 mutant knock-in mouse

A *Ptprz* Pro (1998) to termination (ΔD2) knock-in mouse (Accession No. CDB1127K: http://www2.clst.riken.jp/arg/mutant%20mice%20list.html) was generated as follows: Targeting gene sequences were isolated from a C57BL/6J BAC library (BACPAC) using a homologous recombination approach (Red/ET Recombineering, Gene Bridges) and then inserted into a DT-A-pA/loxP/PGK-Neo-pA/loxP vector (LARGE, RIKEN BDR: http://www2.clst.riken.jp/arg/cassette.html). The mutation of Pro (1998) to termination was performed using a commercial kit (KOD-Plus-Mutagenesis Kit, Toyobo). The positions of the amino acid residues were referred to that of the mouse PTPRZ-A isoform (Genbank; NM_001081306*)*. Genotyping was performed on tail DNAs by PCR. The primers used were (for their positions, see Fig 1A); 5’- GACAGCCACATTCACTCCTATGT -3’ (forward primer) and 5’- TGTCAAAGTTAGTCTAGGATTTCTGACAAC -3’ (reverse primer). PCR products were 319 bp for the wild-type allele and 459 bp for the ΔD2 knock-in allele. PCR was performed with high-yield Taq DNA polymerase (Jena Bioscience), 0.5 μM primer pairs, and 100 ng tail DNAs in 15-μl reactions. Cycling conditions were at 94°C for 180 s, 32 cycles at 94°C for 15 s, 60°C for 30 s, 72°C for 60 s, and a final extension step at 72°C for 420 s.

### cDNA synthesis and quantitative real-time PCR

Total RNA was isolated from brain tissues with TRIzol Reagent kit (Thermo Fisher Scientific). cDNAs were synthesized using the PrimeScript RT reagent kit with the gDNA Eraser (Takara Bio), and used as a template for real-time PCR using a commercial kit (TaKaRa One Step SYBR, Takara Bio) on a real-time PCR system (StepOnePlus Real-Time PCR System). The relative mRNA expression levels of the *Ptprz-A*, *-B*, and *-S* isoforms and ΔD2 mutant isoforms were estimated and normalized to that of glyceraldehyde-3-phosphate dehydrogenase (*Gapdh*). The PCR primers used and sizes of the amplified products are shown in S1 Fig.

### Western blotting

Western blotting experiments of brain extracts using anti-PTPRZ-S [17] and anti-RPTPß (BD Biosciences Cat# 610180,) was performed as described previously [29].

### Open field and novel object exploration tests

Mice were placed in the center circle of a round field (internal diameter, 75 cm and wall height, 40 cm) divided by a grid into twenty-five equal segments under normal lighting conditions (approximately 240 lux at the center area of the open field), and their behaviors were observed for 5 min per day over 3 consecutive days. Horizontal locomotor activity was examined by counting the number of crossings over boundary lines. On the day after the open-field test, the novel object test was started in the same open field. Mice stayed in the open field without objects for 9 min, a white plastic cube (5×5×5 cm) was then placed in the central area, and their exploratory behavior (the number of crossings over the center circle) was recorded for a further 9 min.

### Inhibitory avoidance test

Training and testing were performed in an apparatus with two compartments (15×15×15 cm each): a transparent plexiglas box and a black plexiglas box, separated by a black-colored guillotine door, which was set in a behavioral analyzing system (SCANET, Melquest). The floor was a stainless-steel rod grid comprised of twenty bars with a diameter of 2 mm, which were electrified with a shock generator scrambler (NS-SG01, Neuroscience). The conditioning apparatus was located in a separate room with low levels of background noise produced by ventilation. Illumination was provided by a 36 W light bulb 30 cm above the apparatus.

On the day before the test, mice were allowed to move freely between the two compartments for 30 min for habituation to the conditioning apparatus. Mice were initially placed in the light compartment and this was followed by opening the door. When mice entered the dark compartment, the door was immediately closed, a 1-sec, 0.5-mA footshock was applied after 10 sec, and mice were left there for 2 min. This sequence was repeated until each mouse remained in the light compartment for more than 3 min (cut-off time) when newly placed there. The escape latency of the first trial before receiving the footshock was recorded as the pre-conditioning value. After 24 hrs, the retention of the aversive memory was assessed by measuring escape latency from the light to dark compartment (two trials per session).

### METH-induced locomotor response

METH (Methamphetamine hydrochloride, Dainippon Pharmaceutical) was dissolved to 0.1 mg/ml (for 1 mg per kg per injection of METH) with sterilized 0.9% NaCl (normal saline, Otsuka Pharmaceutical) and injected subcutaneously into the backs of mice. Locomotor activity was measured in a clear acryl chamber (40×26.5×40 cm) with an activity monitoring apparatus (SCANET SV-40, Melquest) with illumination (36 W light bulb 30 cm above).

### *In vivo* microdialysis

Mice were anesthetized with 2% vaporized isoflurane during surgery. An intracerebral guide cannula (CMA 11, CMA/Microdialysis AB) was then stereotaxically implanted on the left nucleus accumbens at a depth of 4.0 mm (coordinates with respect to the bregma: 1.4 mm anterior and 0.8 mm lateral). The guide cannula was secured with dental cement (GC Fuji I, GC Corporation) and then closed with a dummy cannula. Lidocaine cream was applied to the incision site after surgery for acute pain relief. After allowing at least 3 days of recovery from surgery, the dummy cannula was removed and a dialysis probe (membrane length of 1 mm, 6 kDa cut-off, CUP 11, CMA/Microdialysis AB) was set through the guide cannula. The probe was perfused with Ringer solution (147 mM Na^+^, 4 mM K^+^, and 155.6 mM Ca^2+^) at a flow rate of 2.0 μl/min. Microdialysis was performed in awake and unrestrained mice, and the amounts of dopamine in dialysates were measured using online HPLC coupled to an electrochemical detector system (BMA-300, Eicom) according to the manufacturer’s instructions.

### Statistical analyses

Statistical analyses were performed using IBM SPSS Statistics 25 software (SPSS) together with Microsoft EXCEL (Excel for Mac version 16.16.2, Microsoft). Since ANOVA assumes the homogeneity of variance across all conditions, Mauchly’s test of Sphericity was adopted to examine the sphericity assumption within repeatedly measured data; when this test was significant, a Greenhouse-Geisser correction was applied. In Figs 2 and 4, statistical outliers were determined using GraphPad’s QuickCalc Grubb’s test (http://graphpad.com/quickcalcs/grubbs1) with significance set to α = 0.05, and the trimmed data sets were then analyzed by two-way mixed design ANOVA.

## Supporting information

S1 Fig DNA sequencing data of cDNA clones prepared form Δ*D2*/Δ*D2* mice, and primer sets for quantitative real-time PCR analyses. **(A)** cDNA was prepared from Δ*D2*/Δ*D2* mouse brains, was the knock-in region was amplified using forward primer, 5’-GTCAACATATTTGGCTTCTTAAAG-3’ and reverse primer, 5’-GTTACACTGCTTCAGGGCTGTGGAGTAG-3’. The PCR band (271 bp) excised, subcloned into pBluescript vector, and then sequenced. The figures are representative sequencing data of five independent clones. **(B)** Schematic representation of *Ptprz-A* and *Ptprz-B* with their mutant ΔD2, and *Ptprz-S* isoforms. Arrows in the upper part indicate the PCR primer sets designed. Their sequences are as follows: primer set 1 (*Ptprz-A* and its Δ*D2* mutant); forward primer, 5’-CAGGAGTATCCAACAGTTCAGAG-3’ and reverse primer, 5’-CTTCTCAGACTC CAACCCCTC-3’ (amplicon size, 89 bp). Primer set 2 (*Ptprz-B* and its Δ*D2* mutant); forward primer, 5’-CCTCCAGACCACTTGATTTG-3’ and reverse primer for *Ptprz-A* (amplicon, 134 bp). Primer set 3 (*Ptprz-S*); forward primer, 5’-AACCAGAAC GTTCAACCATTTG-3’ and reverse primer, 5’-GAATAGGAATTAGTAACAAC-3’ (amplicon, 138 bp). In the present study, we also used the primer set for control *Gapdh*: forward primer, 5’-ATGGTGAAGGTCGGTGTG-3’ and reverse primer, 5’-GTCGTTGATGGCAACAATC-3’ (amplicon, 99 bp).
(TIF)

S2 Fig Expression levels of TH proteins. Striatal tissue extracts were analyzed by Western blotting using anti-TH antibody (AB152, Merck). The scatter plot shows the signal intensity of TH staining relative to that from the wild-type mice, in which each circle corresponds to an independent experiment (*n* = 6 per group). There were no significant effects of genotype (*F*_(3, 20)_ = 0.703, *P* = 0.561) by UNIANOVA.
(TIF)

## Abbreviations

CAH: Carbonic anhydrase
CNTN1: F3/contactin
DAT: Dopamine transporter
Gapdh: Glyceraldehyde-3-phosphate dehydrogenase
KO: Knockout
METH: Methamphetamine
PTKs: Protein tyrosine kinases
PTN: Pleiotrophin
PTPRZ: Protein tyrosine phosphatase receptor type Z
PTPs: Protein tyrosine phosphatases
RPTPs: Receptor-like PTPs
SDC3: Syndecan3
TH: Tyrosine hydroxylase
