# Supplementary figures and images for: Behavioral and neurological analyses of adult mice carrying null and distinct loss-of-receptor function mutations in protein tyrosine phosphatase receptor type Z (PTPRZ)
Source: PLoS One. 2019 Jun 13;14(6):e0217880. doi: 10.1371/journal.pone.0217880 (PMC6563982; doi:10.1371/journal.pone.0217880)

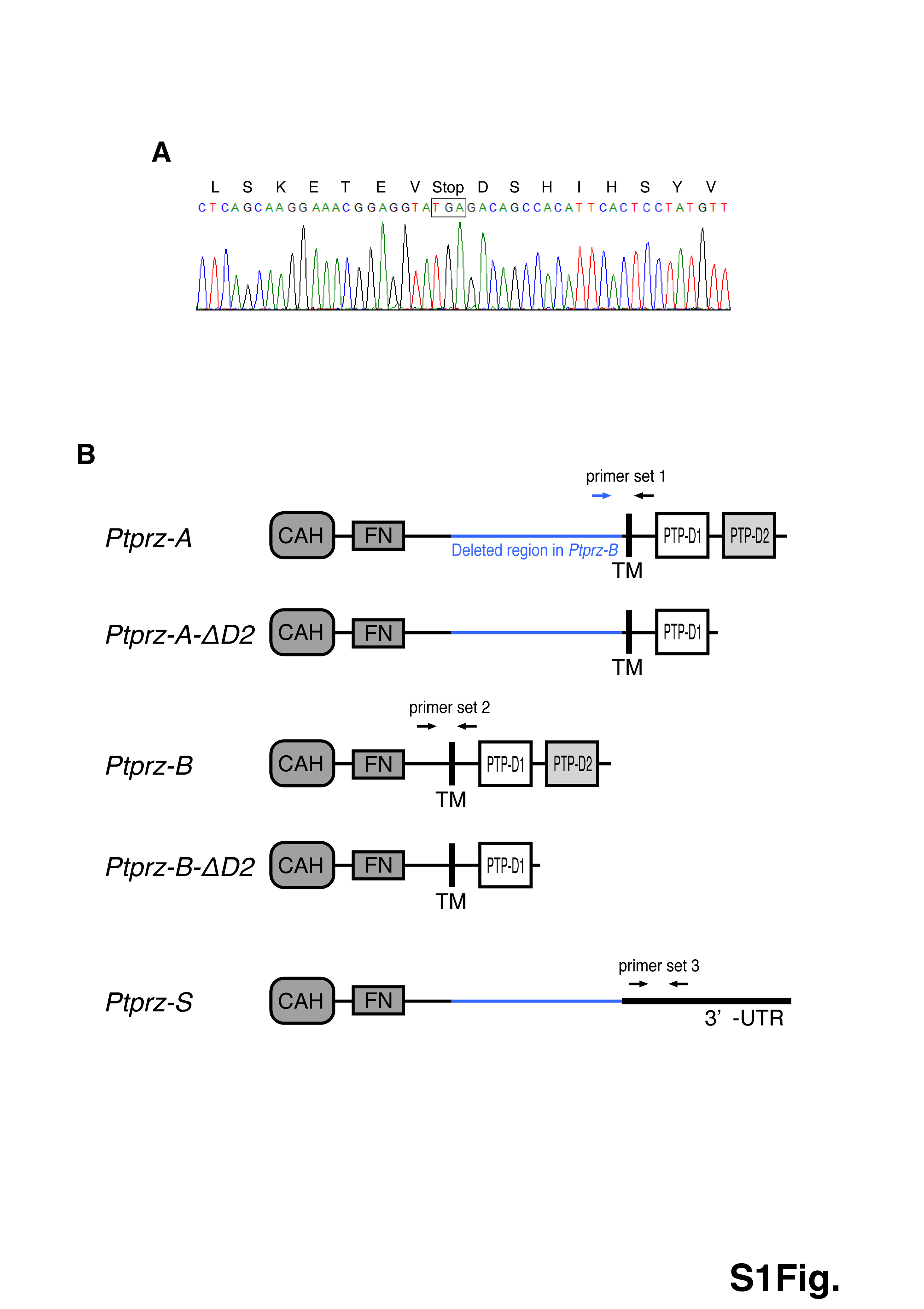

Supplement: S1 Fig — (A) cDNA was prepared from ΔD2/ΔD2 mouse brains, was the knock-in region was amplified using forward primer, 5’-GTCAACATATTTGGCTTCTTAAAG-3’ and reverse primer, 5’-GTTACACTGCTTCAGGGCTGTGGAGTAG-3’. The PCR band (271 bp) excised, subcloned into pBluescript vector, and then sequenced. The figures are representative sequencing data of five independent clones. (B) Schematic representation of Ptprz-A and Ptprz-B with their mutant ΔD2, and Ptprz-S isoforms. Arrows in the upper part indicate the PCR primer sets designed. Their sequences are as follows: primer set 1 (Ptprz-A and its ΔD2 mutant); forward primer, 5’-CAGGAGTATCCAACAGTTCAGAG-3’ and reverse primer, 5’-CTTCTCAGACTC CAACCCCTC-3’ (amplicon size, 89 bp). Primer set 2 (Ptprz-B and its ΔD2 mutant); forward primer, 5’-CCTCCAGACCACTTGATTTG-3’ and reverse primer for Ptprz-A (amplicon, 134 bp). Primer set 3 (Ptprz-S); forward primer, 5’-AACCAGAAC GTTCAACCATTTG-3’ and reverse primer, 5’-GAATAGGAATTAGTAACAAC-3’ (amplicon, 138 bp). In the present study, we also used the primer set for control Gapdh: forward primer, 5’-ATGGTGAAGGTCGGTGTG-3’ and reverse primer, 5’-GTCGTTGATGGCAACAATC-3’ (amplicon, 99 bp). (TIF) [file pone.0217880.s001.tif]

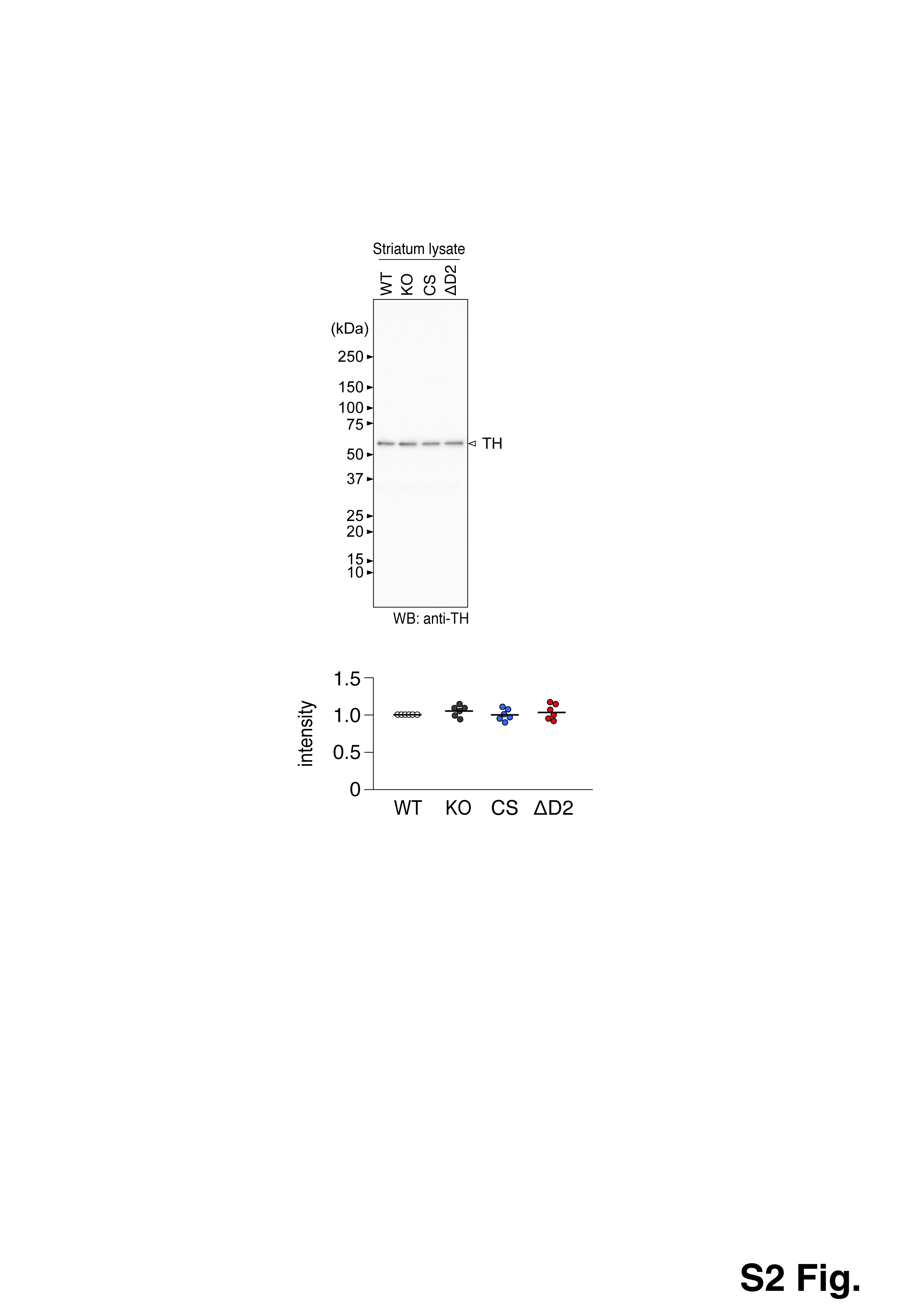

Supplement: S2 Fig — Striatal tissue extracts were analyzed by Western blotting using anti-TH antibody (AB152, Merck). The scatter plot shows the signal intensity of TH staining relative to that from the wild-type mice, in which each circle corresponds to an independent experiment (n = 6 per group). There were no significant effects of genotype (F(3, 20) = 0.703, P = 0.561) by UNIANOVA. (TIF) [file pone.0217880.s002.tif]
